# Delivery of healthcare and healthcare education in the digital era and beyond: opportunities and considerations

**DOI:** 10.4069/kjwhn.2023.09.06

**Published:** 2023-09-26

**Authors:** Seon Yoon Chung

**Affiliations:** College of Nursing, University of Wisconsin Oshkosh, WI, USA

## Introduction

The coronavirus disease 2019 (COVID-19) pandemic brought unprecedented challenges to societies worldwide. Countries, industries, and the general public had to adjust their operations to accommodate imposed restrictions or requirements, such as physical distancing and sanitization. Healthcare agencies had to pivot promptly to address exponentiating healthcare needs, including vaccination, treatment, and management of COVID-19-related illness. Schools and workplaces had to swiftly find alternative modes for delivering instruction and to continue offering their services. These circumstances allowed widespread utilization of the innovative technologies from the fourth industrial revolution [1] (i.e., Industry 4.0). This article outlines changes observed in the healthcare and healthcare education settings in the digital era and the subsequent opportunities and considerations.

## Healthcare settings

In clinical practice settings, telehealth or telemedicine has been highlighted as a solution for conducting remote assessment and consultation during times of physical isolation. In early 2020, the American College of Obstetricians and Gynecologists made recommendations for providers to consider increasing familiarity with telehealth, and hospitals adopted telehealth to care for high-risk obstetric patients [2,3]. As part of their Global Strategy on Digital Health 2020–2025, the World Health Organization defines telemedicine as the delivery of healthcare service(s) by healthcare professionals using telecommunication technologies for the exchange of information aimed at advancing the health of individuals and communities [4]. Telehealth utilizes innovative technologies such as web or videoconferencing, social communication apps, and other communication media for assessment, diagnosis, treatment, and prevention of illness and injuries. Other technologies supporting telehealth’s convenience include biomedical sensors that measure biometrics, and fifth-generation (5G), and Wi-Fi networks that allow for the information to be stored and accessed in the cloud.

Beyond the benefits of reducing potential exposure to viruses during in-person visits, telehealth can promote access to healthcare by eliminating the need for a mode of transportation or the time needed to travel [5]. This can be liberating for individuals with mobility restrictions or caregiver responsibilities. In particular, telehealth can allow childbearing or pregnant women who may have other competing roles and priorities to access their provider at their location of convenience in a timely manner [6]. As an example, Bonciani et al. [7] provided antenatal classes online during the COVID-19 pandemic to support them throughout their maternal care and found this approach to be valuable in reaching more women during pregnancy. Similarly, Álvarez-Pérez et al. [8] created massive open online courses to promote digital health literacy for pregnant and lactating women in Europe. A recent systematic review reported that the use of patient-centered decision support tools enabled through digitalization and new technology increased pregnant women’s knowledge and satisfaction regarding maternity care [9]. A telehealth lifestyle intervention was also studied to reduce excess gestational weight gain in overweight or obese pregnant women, suggesting its potential utility in improving healthy behaviors in this population [3].

In addition to the potential benefits of telehealth in promoting pregnant and lactating women’s knowledge, satisfaction, or health behaviors, burgeoning evidence suggests the effectiveness of telehealth in supporting women’s mental health. Koç et al. [10] conducted a systematic review and reported evidence of the effectiveness of telehealth on the mental health of women with breast cancer, such as reduced symptoms of depression, anxiety, and fear of relapse as well as improved cognitive function and psychological strengths. Similarly, a recent meta-analysis of women with postpartum depression showed significantly lower scores of anxiety and Edinburgh Postnatal Depression Scale in the telehealth group than in the control group [11]. Other obstetric and gynecologic health outcomes found to be improved by telehealth interventions include fewer scheduled outpatient visits in the case of high-risk obstetrics, early access to medical abortion services, and higher oral contraception rates [12]. The use of artificial intelligence (AI) to promote preventive interventions in areas where conditions and outcomes are sex- and gender-based, such as risk-screening for cardiovascular disease, is another area to be further explored [13].

Though the pandemic accelerated the utilization of advancements from the fourth industrial revolution, technologies such as robotics were already in use prior to the pandemic, especially in healthcare settings. Based on a cohort study of 169,404 patients in 73 hospitals in the United States, Sheetz et al. [14] reported a rising trend in the use of robotic surgery for all general surgery procedures, from 1.8% in 2012 to 15.1% in 2018. In Korea, Ryu et al. [15] reported robot-assisted nipple-sparing mastectomy as a feasible and acceptable surgical technique. For early-stage cervical cancer, Alfonzo et al. [16] found that there is no survival difference between robotic and open radical hysterectomy, based on a nationwide population-based cohort study in Sweden.

The rapid advancement of technology in the practice setting justifies healthcare providers in incorporating those technologies in a healthcare context and guiding clients to utilize those resources, as applicable. Simultaneously, it is important to understand the challenges inherent to technology-enhanced solutions such as telehealth, notably the digital divide and limited access to internet or telehealth devices; these limitations should be carefully considered to ensure health equity [17,18]. This leaves healthcare educators to consider the expanded definition of practice-ready graduates as well as the competencies required to be successful in the rapidly evolving high-tech healthcare environment.

## Healthcare education settings

Prior to the pandemic, traditional education in healthcare was characterized by in-person lectures, labs, and clinical experiences. Seasoned educators would agree that hands-on experiential learning in person was by far the most common instructional method. In the early phases of the COVID-19 pandemic, when social gatherings including campus activities and classroom instructions were prohibited, educators had to transition courses online over a very short period of time. Traditional lectures were delivered online synchronously using web or videoconferencing technology such as Zoom or Microsoft Teams [19]. Some lectures were recorded and uploaded to learning management systems such as Blackboard and Canvas, and students were granted access to watch them asynchronously. The latter option was also used as an alternative offering to students who might not have access to high-speed internet or those who had to miss synchronous sessions due to illness or childcare responsibilities.

When students were not allowed in clinical settings but were allowed in labs on campus, in-person clinical simulations using technologies such as high-fidelity manikins were used to supplement clinical teaching and learning. When students were not allowed on campus, remote/distance simulations employing web conferencing technologies were conducted in which instructors or actors served as standardized patients. This allowed students to care for the patient in a given scenario [19].

As companies started to release more online simulation products using AI (e.g., AI chatbot), educators were empowered to provide their students with more opportunities to practice and demonstrate their understanding and competence. The AI-powered simulations allow students to have a dialogue and foster their communication skills [20].

More recently, immersive technology using wearable devices has become more accessible in the educational arena. Students can now use headsets or head-mounted displays to enter a virtual, augmented, or mixed-reality setting and engage with the environment using kinesthetic haptic devices [21]. Universities and companies have leveraged this technology to create scenario-based virtual simulations [20]. The simulations allow students to practice clinical reasoning in two-dimensional as well as three-dimensional environments. They also let students demonstrate not only their understanding, through answering exam questions or writing care plans, but also their ability to apply it to a given situation. Technology can enrich students’ learning experiences by providing additional exposure to diverse scenarios in a safe and immersive environment. These advances invite higher education administrators to reconsider the role that their institutions can play in graduating students who are truly prepared to enter fast-evolving industries.

Many schools of nursing are building culture and infrastructure to promote innovation and to position not only their students but others’ to lead innovations in health systems [22]. As an example, the University of Pennsylvania’s School of Nursing offers an online open-access platform with resources to yield innovative solutions for problems in healthcare [23]. Universities or colleges equipped with the awareness and tools to expose students to future possibilities, with educators who are nimble and open to leading change, and with the financial capacity and a solid foundation to encourage innovation, may find themselves in a leading position.

## Opportunities and considerations

High-quality information and content are saturated and available on demand, through platforms such as Google, YouTube, Apple, Netflix, or Hulu. Animations feature advanced technology—software and hardware—that is personalized and deeply incorporated into daily lives. A 2021 movie titled *Ron’s Gone Wrong* features a personalized robot companion that is portrayed as integral to school-age students’ social lives, similar to having a smartphone. Even a movie released in 2014 titled *Big Hero 6* features Baymax, a personalized healthcare companion robot with the capacity to provide treatment based on assessment as well as to care and be a person’s closest friend [24]. Since then, *Big Hero 6* was turned into a television series, and in summer 2022, *Baymax*! premiered as its own series [25,26]. Baymax is also referred to as a “nurse/robot” [27].

Generation Z, currently entering higher education and the workforce, grew up watching these movies and television series and having access to AI agents such as Apple’s Siri and Amazon’s Alexa. Generation alpha, amid or entering K-12 education, is growing up with further evolved AIs, such as ChatGPT and DALL-E2 [28,29]. The value of higher education and professionals is being questioned, since news articles have reported exam types for which ChatGPT performed at or near the passing threshold, including the United States Medical Licensing Exam and bar exams, as well as the Scholastic Aptitude Test, Graduate Record Examination, USA Biology Olympiad, and a range of Advanced Placement examinations [30,31].

At minimum, this calls for educators to reflect on our practices and consider the need to emphasize higher-order thinking. What do you do with the information, and what questions do you ask to gather the information needed to solve problems and achieve the goal at hand? In the United States, the National Council of State Boards of Nursing launched the Next Generation National Council Licensure Examination for Registered Nurses examination in April 2023, which emphasizes clinical judgment skills as an essential skill for nurses to demonstrate [32,33]. The American Association of Colleges of Nursing released guidelines on essential competencies for nursing education in spring 2021 [24]. Colleges of nursing have begun to shift towards a competency-based education model and incorporate technology as supplemental learning tools in the curriculum to promote opportunities to practice application and demonstrate competencies.

Healthcare administrators are also called upon to explore the implications of emerging technology and the evolving expectations of our healthcare consumers and the incoming workforce [34]. Some hospitals have already begun to implement telehealth. Telehealth is supported by Medicaid, Medicare, and commercial healthcare plans in many states in the United States [35-37]. More companies and even public schools are considering offering spaces that allow their employees to seek telehealth visits without having to leave their workplace. This means that the point of care could shift to our communities and homes. The role nurses play in care coordination beyond the acute care setting would become more emphatic. Exposure to the paradigm shift, the changing role of nurses, and the subsequent need to reflect these changes by educating practice-ready nurses is vital.

For both education and healthcare, the core value may lie within personalized and person-centered approaches. It would not be surprising to see a healthcare consumer preferring care that meets their needs, fits their lifestyle, and has an interdisciplinary team collaborating and utilizing enhanced technology specifically to meet clients’ needs. A team of healthcare professionals who can provide personalized holistic care would be undoubtedly welcomed. Similarly, students will likely prefer education that meets their needs, fits their lifestyles, and has a specialized student success team collaborating and utilizing enhanced technology to provide student-centered, personalized, holistic support.

Some important considerations arise as we look into embracing technology developments and their utilization in the education and healthcare industries. For providers of service-oriented education or healthcare, the cost to build and maintain infrastructure for advanced technology cannot be disregarded and should be carefully budgeted for. It is also important to keep health and education equity at the forefront in making decisions so that consumers are granted equal access to the services provided.

As more private data is collected, stored, and transmitted, all parties involved need to be vigilant about information privacy and cybersecurity. Face and voice recognition, used commonly now, could be dangerous if misused in combination with machine-learning technology. The ever-larger number of devices and cloud services used by individuals could provide additional entry points for security breaches. While it comes with its own limitations, blockchain technology may be used to promote the security and privacy of sensitive information protected by the Health Insurance Portability and Accountability Act, to leverage its features such as decentralization, immutability, transparency, and traceability [38].

The general public’s level of comfort in sharing their personal information and measures to accommodate various levels of preference should be considered. For example, in a survey recently conducted in Sweden by Belfrage et al. [39], the general public’s trust in the ability of healthcare to protect electronic patient data was high (81.9%). There were individuals with low levels of trust, however, who preferred to be asked for permission before their personal data could be used and who were less open to allowing it.

It would be prudent to consider current and future providers’ perceptions and their needs as well. A mixed methods study conducted in Germany involving 80,000 medical students showed that most students reported a positive attitude towards digital applications in medicine. Thirty-eight percent of the students did not feel ready to answer questions related to AI because it was not formally covered in the curriculum, suggesting a need to incorporate digital content into the curriculum [40]. A cross-sectional survey of gynecologists in Germany showed that 67.3% of respondents would like to use telemedicine, 73.2% would use it during follow-up at the treatment phase, and 51.5% would opt for telecounseling to improve care [41].

Jarva et al. [42] reported that healthcare professionals perceive digital health competence to be focused on the ability to provide patient-centric care by evaluating the feasibility of using digital health services jointly with more traditional methods. Opportunities should be available to continue improving our understanding of the changing landscape of digital healthcare and the roles that healthcare providers play in this environment. Conversely, opportunities should be offered for healthcare providers to provide feedback and shape how technology can best be leveraged in educational and healthcare settings.

## Conclusion

Advanced communication and connectivity technologies provide exciting, geographically boundless opportunities to promote global collaboration. The innovative technologies of Industry 4.0 are only forecasted to grow. The COVID-19 pandemic forced many around the globe to be flexible and open-minded, to think outside-the-box, and be innovative thinkers to overcome the enormous range of challenges that it brought us. While technology comes with its own concerns, it has demonstrated its positive utility in securing access to education and healthcare.

South Korea is referred to as an innovative, high-technology society with a strong infrastructure, such as pervasive 5G wireless technology [43,44]. Smart technology is well integrated into daily activities, and the general public has access to affordable internet and technology [45-47]. This infrastructure presents an opportunity for the country to lead in education and healthcare in the digital era and beyond. While there is no one-size-fits-all solution to the delivery of healthcare and healthcare education in the digital era, there is no question that technology could be leveraged to address certain barriers to healthcare and healthcare education. By the same token, several aspects, such as digital literacy and the digital divide, need to be carefully considered to prevent unintended consequences. Having a clear end goal—the promotion of health and well-being of our students and patients—will keep us moving in the right direction.
